# Enhancing 3D Models with Spectral Imaging for Surface Reflectivity

**DOI:** 10.3390/s24196352

**Published:** 2024-09-30

**Authors:** Adam Stech, Patrik Kamencay, Robert Hudec

**Affiliations:** Department of Multimedia and Information-Communication Technologies, University of Zilina, 010 26 Zilina, Slovakia; adam.stech@feit.uniza.sk (A.S.); robert.hudec@feit.uniza.sk (R.H.)

**Keywords:** photogrammetry, Agisoft Metashape, SIFT, SURF, SfM, 3D reconstruction, surface reflectivity

## Abstract

The increasing demand for accurate and detailed 3D modeling in fields such as cultural heritage preservation, industrial inspection, and scientific research necessitates advanced techniques to enhance model quality. This paper addresses this necessity by incorporating spectral imaging data to improve the surface detail and reflectivity of 3D models. The methodology integrates spectral imaging with traditional 3D modeling processes, offering a novel approach to capturing fine textures and subtle surface variations. The experimental results of this paper underscore the advantages of incorporating spectral imaging data in the creation of 3D models, particularly in terms of enhancing surface detail and reflectivity. The achieved experimental results demonstrate that 3D models generated with spectral imaging data exhibit significant improvements in surface detail and accuracy, particularly for objects with intricate surface patterns. These findings highlight the potential of spectral imaging in enhancing 3D model quality. This approach offers significant advancements in 3D modeling, contributing to more precise and reliable representations of complex surfaces.

## 1. Introduction

Photogrammetry [1,2] is a sophisticated technique used to create accurate 3D models of objects or structures by analyzing and processing multiple 2D images taken from different viewpoints. The core principle behind photogrammetry is triangulation, where common points are identified across two or more images. By analyzing these points, the software calculates the position, orientation, and shape of the object in 3D space. Modern photogrammetry leverages powerful algorithms and computer vision techniques to automate much of this process, allowing even complex surfaces and structures to be reconstructed with high accuracy. The process can be broken down into several steps, starting with image capture, followed by image alignment, point cloud generation, mesh creation, and texture mapping. Photogrammetry does not require special sensors, and its ability to work with off-the-shelf cameras makes it highly accessible and cost-effective for many industries. Digitalization is advancing rapidly, with modern methods like photogrammetry and laser scanning becoming increasingly effective. By integrating image processing algorithms such as Structure from Motion (SfM), Multi-View Stereo (MVS), Scale-Invariant Feature Transform (SIFT), and Speeded-Up Robust Features (SURF), photogrammetry has achieved greater accuracy and no longer requires specialized cameras for image capture. Through the use of photogrammetry, professionals can create accurate 3D models for analysis, visualization, or preservation, offering a versatile tool that bridges the gap between digital and physical worlds [1,2].

An example of the application of photogrammetry in the medical field is its use in the sequential imaging process for the production of prosthetic devices. Photogrammetry enables the creation of highly detailed and accurate 3D models of a patient’s anatomy, which can be valuable for designing custom-fit prostheses. While alternative methods, such as 3D scanning and CAD-based modeling, are often faster and more precise, photogrammetry offers a more accessible and cost-effective approach in certain contexts. For instance, in a study conducted by the research group of Taqriban et al. [3], the photogrammetry method was employed to create a dataset using four commercially available cameras. This method allowed them to capture detailed images from various angles, which were then processed to generate a 3D model of the target anatomy. Although photogrammetry may not always match the speed and precision of other methods, it presents a viable option in scenarios where accessibility, cost, or specific imaging requirements are factors. That study demonstrated that photogrammetry could be adapted for medical purposes, particularly in resource-limited settings, and could serve as a supplementary tool for prosthetic design and production.

Another example of the implementation of photogrammetry is its integration with laser scanning technology. This combination enhances the accuracy of the captured data but typically increases the time required for data acquisition. By merging these two techniques, the resulting 3D model provides a more precise and detailed representation of the object compared to using photogrammetry or laser scanning alone. In a study by Malhotra et al. [4], that research group proposed an automated solution for room digitization by coupling photogrammetry with LiDAR-based laser scanning technology. In their study, a drone equipped with both a camera and Time-of-Flight (ToF) technology, operating on the LiDAR principle, was utilized. This system measured the distance between the sensor and the object by calculating the time it took for a transmitted signal to be reflected back to the sensor. The UAV technology enabled the automation of the photogrammetric process, determining the optimal number of frames needed to create an accurate 3D model of the room. This integration of photogrammetry and laser scanning provided a robust, automated approach for digitizing indoor environments, offering highly accurate spatial data that could be applied in fields such as architecture, construction, and interior design. The automated frame selection enhanced efficiency, reducing manual intervention and ensuring a more streamlined workflow in creating precise 3D representations.

The photogrammetric procedure can be modified by changing the light conditions from the normal process, which allows the definition of spectral photogrammetry. This approach was applied by the group of Mathys et al. [5], which focused on the digitization of biological objects with predominantly reflective surfaces [6,7]. The digitization of these objects was performed using two cameras that captured different wavelengths of the visible spectrum, as well as infrared and ultraviolet radiation, using different polarization filters for each wavelength. When capturing an object using other methods such as structured light, a problem arises when creating a solid model, particularly for the object used in our case (butterfly). Since the object represents a negligible depth, it is only possible to create a 3D model in the view plane. The photogrammetric procedure requires some knowledge in imaging and dataset creation, which is the most important part of the visualization and subsequent processing into a 3D model. In the case of digitization, it must be assumed that the object of interest for digitization is not homogeneous. With the advent of digital photography and advanced software, modern photogrammetry has become more accessible and precise. Tools such as Unmanned Aerial Vehicles (UAVs), high-resolution digital cameras, and powerful photogrammetry software enable automated image processing and 3D reconstruction. Software applications like Agisoft Metashape 1.8.1, Pix4Dmapper 4.9.0, and Autodesk ReCap 23.1.2.396 are widely used to process images and generate 3D models. A novel compressive 3D imaging spectrometer based on the coded aperture snapshot spectral imager (CASSI) was proposed in [8]. The 3D spatio-spectral sensing process was modeled by integrating computational integral imaging with compressive coded aperture spectral imaging.

The field of 3D modeling has seen significant advances with the integration of spectral imaging techniques, particularly in the enhancement of surface reflectivity. Spectral imaging, which captures image data over different wavelengths of light, provides detailed information about the material and surface properties of objects. This integration has opened new avenues for creating highly accurate and visually rich 3D models. The primary benefit of using spectral imaging in 3D modeling is the enhancement of surface detail and reflectivity. This is critical for applications where accurate surface representation is essential, such as cultural heritage preservation, medical imaging, and industrial inspection. For example, in cultural heritage, spectral imaging can reveal underdrawings and restorations on artifacts, providing invaluable information for conservation efforts [9,10,11].

Recent advances have focused on integrating spectral imaging with 3D scanning technologies. By combining spectral data with 3D spatial data, researchers can create models that have not only accurate geometric information, but also detailed surface reflectivity properties. This integration enables the creation of models that more accurately simulate real-world lighting conditions, enhancing visual realism. Advanced software tools such as Cloud Compare have been used to evaluate the accuracy of 3D models created with spectral imaging data. These evaluations compare spectral imaging-enhanced models with reference models created using high-precision methods such as digital radiography. The results consistently show lower error rates and higher fidelity in the spectral imaging-enhanced models [12,13].

The goal of this research was to establish a comprehensive methodology that integrates spectral imaging data to enhance the quality and precision of 3D models. By incorporating spectral imaging, the research aimed to achieve improved surface detail and enhanced reflectivity in 3D reconstructions, capturing fine textures and subtle variations that are often missed by traditional methods. Additionally, the study sought to develop a robust framework that seamlessly combines spectral imaging with conventional 3D modeling techniques. This approach has the potential to advance the field of 3D modeling by offering more precise, reliable, and versatile methods for capturing intricate surface features, with applications ranging from cultural heritage preservation to industrial design and scientific research. Furthermore, the integration of spectral imaging could open new avenues for analyzing material properties and surface characteristics, pushing the boundaries of current 3D modeling technologies.

## 2. Materials and Methods

This section outlines the materials and methods required to implement a systematic approach to performing Iterative Closest Point (ICP) registration using Cloud Compare. By following these steps, highly accurate alignment of three-dimensional point clouds can be achieved, facilitating various applications in fields such as three-dimensional modeling, robotics, and computer vision. The methodology of the study emphasizes the importance of careful parameter selection, preprocessing, and iterative refinement to ensure the accuracy and efficiency of point cloud data registration. By leveraging the capabilities of ICP and Cloud Compare, practitioners can achieve highly accurate 3D reconstructions, contributing to advances in fields ranging from cultural heritage to autonomous navigation. As the technology continues to evolve, ongoing research and development of ICP algorithms and 3D data processing tools such as Cloud Compare will continue to improve their accuracy, efficiency, and applicability, driving innovation in many fields.

### 2.1. ICP Registration and Cloud Compare

Iterative Closest Point (ICP) registration is a powerful algorithm that is widely used in the field of 3D data processing. The primary objective of ICP is to align two 3D point clouds, designated as the source and target, respectively, by iteratively minimizing the distance between corresponding points. This method is of great importance in a number of fields, including 3D modeling, robotics, computer vision, and augmented reality, where the precise alignment of 3D data is of the utmost importance [14,15]. ICP is a process that follows a series of steps designed to progressively improve the alignment of the point clouds [14,15]:

1. Initial alignment: a rough initial alignment is provided, either through manual positioning or automatic feature-based methods.
2. Correspondence estimation: For each point in the source cloud, the nearest point in the target cloud is identified. This creates a set of point correspondences.
3. Transformation computation: using the correspondences, the optimal transformation (rotation and translation) that minimizes the mean squared error between the source and target points is computed [14].
4. Iteration: steps 2–4 are repeated until convergence, i.e., until the changes in the alignment fall below a predefined threshold or a maximum number of iterations is reached [15].

Cloud Compare is particularly valued for its user-friendly interface and robust performance, making it a popular choice among researchers and practitioners in the field of 3D data processing. Cloud Compare [16] integrates ICP registration as one of its core functionalities [17]. Here is how ICP registration is typically performed within the software:

1. Initial alignment: Users can perform a rough initial alignment using manual transformation tools or automated feature-based methods provided by the software.
2. Applying ICP: Cloud Compare offers a straightforward interface to apply the ICP algorithm. Users can configure parameters such as the number of iterations, convergence criteria, and distance thresholds to tailor the registration process.
3. Post-processing: After registration, Cloud Compare offers various tools for further analysis and processing, such as computing distance maps, merging point clouds, and exporting results.

### 2.2. Proposed Method for Selection of Images

**Figure 1:**
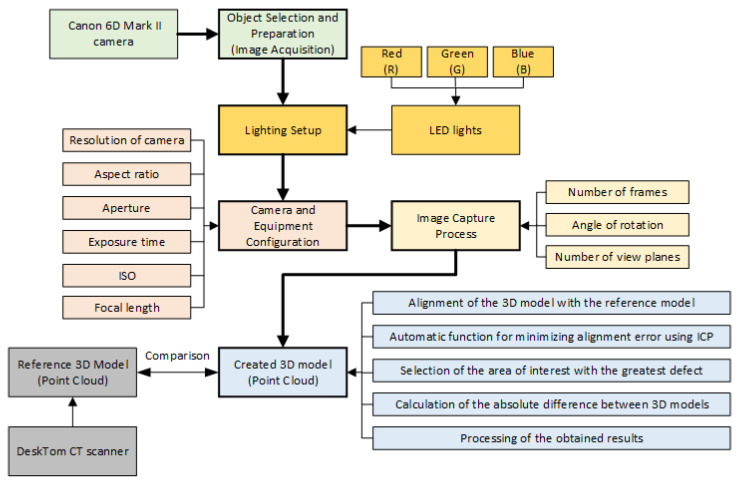
Block diagram for selection of images.

To effectively enhance 3D models with spectral imaging for surface reflectivity, a systematic method for selecting the appropriate images is crucial. The proposed method (see Figure 1) involves several key steps to ensure that the images used are of high quality and provide comprehensive spectral and spatial information:

1. Object selection and preparation.
2. Lighting setup.
3. Camera and equipment configuration.
4. Image capture process.

This proposed method, as is shown in Figure 1, ensures that the selection of images for enhancing 3D models with spectral imaging is systematic and thorough. By focusing on quality assessment, alignment accuracy, and comprehensive coverage, the method aims to produce highly accurate and realistic representations of surface reflectivity. This approach facilitates the creation of enhanced 3D models that are valuable for various applications, from digital heritage preservation to material analysis and computer graphics.

#### 2.2.1. Object Selection and Preparation

The object under consideration is of biological origin, specifically a butterfly. Butterflies are known for their intricate and delicate structures, which provide a unique challenge in macro-photogrammetry. This experiment focused on capturing detailed images of the butterfly, highlighting its distinctive surface features and dimensional complexity. The surface of a butterfly’s wings is covered with tiny scales that contribute to its vibrant colors and patterns. These scales are arranged in overlapping rows, creating a mosaic-like appearance. The colors observed are not solely due to pigments but are also a result of structural coloration, where microscopic structures interfere with light. This phenomenon can cause dramatic color shifts when viewed from different angles, known as iridescence. These changes in color are particularly pronounced in certain regions of the wings, where the scales are structured to reflect light in specific ways.

**Figure 2:**
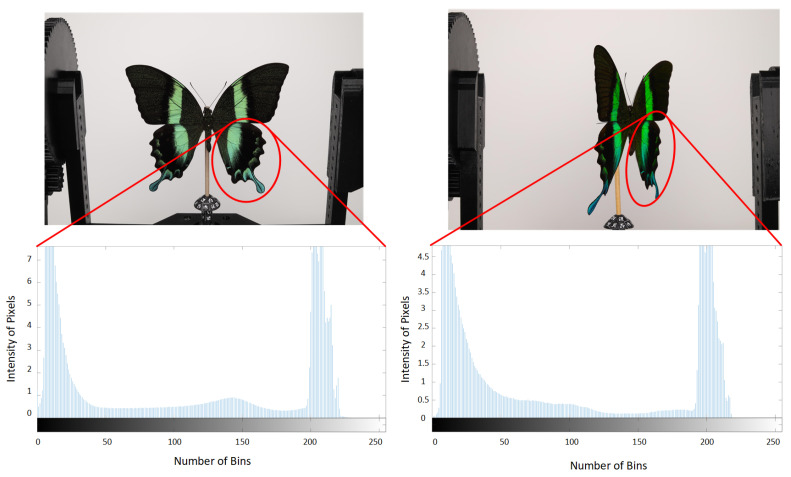
Macro-photogrammetry of a butterfly’s surface showing color changes with tilt.

The dimensional complexity of butterfly wings encompasses curvature, overlapping scales, and the minute details of wing edges and veins. These features necessitate meticulous calibration and positioning throughout the photogrammetry process to guarantee precise capture and reconstruction of the wing surface. Furthermore, the experiment sought to document and analyze color changes caused by tilt. As the angle of light and observation varies, specific regions of the butterfly’s wings display varying hues. This iridescence is most pronounced in species such as the Morpho and Papilio Blumei butterflies, where structural coloration can shift from blue to green to purple depending on the angle. Figure 2 illustrates these remarkable color changes, demonstrating the butterfly’s ability to manipulate light and create stunning visual effects.

**Figure 3:**
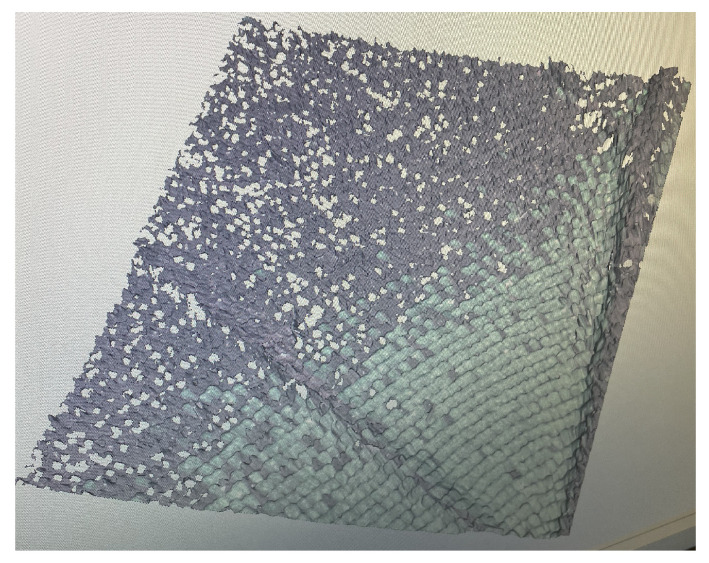
A close-up of a *Papilio blumei* butterfly wing showing microscopic scales that create vibrant, shimmering colors due to structural coloration, resulting in iridescence.

The primary challenges inherent to this experiment include maintaining a delicate equilibrium between capturing high-resolution details and addressing the intrinsic fragility and dynamic nature of the butterfly’s wings. To surmount these challenges, advanced macro-photogrammetric techniques, including focus stacking and precise lighting control, were employed to achieve a comprehensive and detailed representation of the butterfly’s surface. As the object is rotated, a color transformation is observed in the wing area of the butterfly, specifically in the species *Papilio blumei*. This species is renowned for its striking iridescent wings, which display vibrant color changes when observed from different angles. The wing surface of *Papilio blumei* is covered with microscopic scales (see Figure 3), which create structural coloration, resulting in a phenomenon known as iridescence. The iridescence of the wings presents a unique challenge in collecting consistent visual data, as it causes them to change color dramatically.

A histogram is presented for more effective interpretation (the color image has been converted to grayscale as is shown in Figure 2) Converting the color image to grayscale allows for a clearer analysis of the intensity and distribution of light across the wing surface, independent of color variations. This method simplifies the identification of patterns and features by focusing on luminance contrast rather than chromatic information. The observed phenomenon of color transformation can pose significant challenges in aligning and extracting local features from images. As the butterfly’s wings tilt and rotate, the shifting iridescence can cause inconsistencies in feature detection algorithms. These algorithms rely on stable and distinct visual landmarks to accurately map and align images. However, the dynamic color changes can cause these landmarks to become misaligned, complicating the image processing workflow. These challenges become apparent in subsequent processing steps. The color change due to iridescence can cause traditional feature extraction methods, which often rely on consistent color or intensity patterns, to fail or produce errors. Advanced image processing techniques, such as robust feature matching algorithms and adaptive alignment methods, are required to mitigate these effects. In addition, the incorporation of multi-angle imaging and photometric stereo techniques can help capture the full range of color transformations, providing a more comprehensive dataset for analysis. Understanding the behavior of *Papilio blumei* wing coloration under different viewing angles is critical for accurate macro-photogrammetry. By overcoming the challenges posed by iridescence, researchers can improve the accuracy and reliability of 3D models and other analytical results derived from the images used in different visible wavelengths of light. This understanding not only improves the technical aspects of image processing but also contributes to the broader field of optical and structural studies in biological specimens.

#### 2.2.2. Lighting Setup

**Figure 4:**
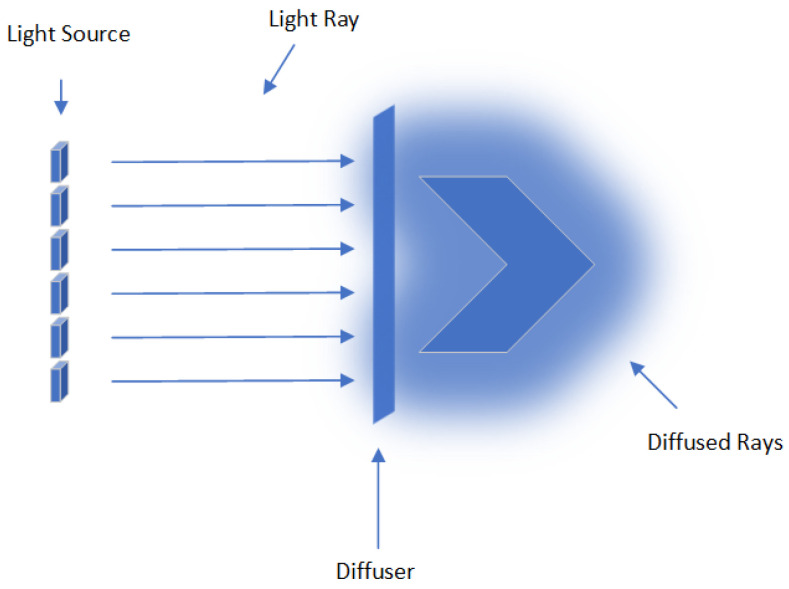
Description of the diffuser.

The object was illuminated using LED lights with diffuse glass as is shown in Figure 4. The use of diffuse glass is a critical component in achieving high-quality imaging, especially when dealing with objects that have complex surface geometries, such as the butterfly *Papilio blumei*. Diffuse glass helps to scatter the light evenly across the surface of the object, thereby reducing the intensity of direct light and minimizing the formation of harsh shadows and dark spots. This is particularly important for objects with folds, creases, or intricate textures, as it ensures that these features are uniformly illuminated and captured in detail.

In the context of macro-photogrammetry, diffuse lighting is essential for several reasons. Firstly, it enhances the visibility of fine details by providing soft, even illumination that highlights the surface features without introducing glare or specular reflections. This is crucial for capturing the macro-structures of the butterfly’s wings, such as the arrangement of patterns. Secondly, diffuse lighting reduces the contrast between different parts of the object, which can otherwise lead to a loss of detail in areas that are either too bright or too dark. By providing a more balanced light distribution, diffuse glass helps in maintaining the dynamic range of the images, preserving details across the entire surface. In addition, the use of LED illumination provides additional benefits in terms of color stability and energy efficiency. LEDs provide a consistent light output without significant fluctuations in intensity or color temperature, ensuring reliable and repeatable imaging conditions. This consistency is essential for accurate color reproduction and maintaining the integrity of the data collected during the photogrammetry process. In addition, LEDs generate less heat than traditional lighting sources, reducing the risk of damage to delicate biological specimens such as butterfly wings during prolonged imaging sessions.

In addition, the color temperature parameter (Table 1) was set on the sensing device to compensate for the object’s color. This ensured that the object’s color was not altered, which was crucial for processing the object and achieving color fidelity in the 3D model creation. The presence of objects with disparate reflectivity can give rise to interference in the formation of the dataset, which may subsequently give rise to issues in the 3D model creation process. Such interference can result in misaligned images or failure to form a 3D model. To address this issue, digitizing with a single light spectrum was avoided. Instead, the white light spectrum was decomposed into its three basic components. In the experiment, where it was necessary to ascertain the specific light spectrum falling on the object, the use of lights that provided this capability (Table 2) was employed.

**Figure 5:**
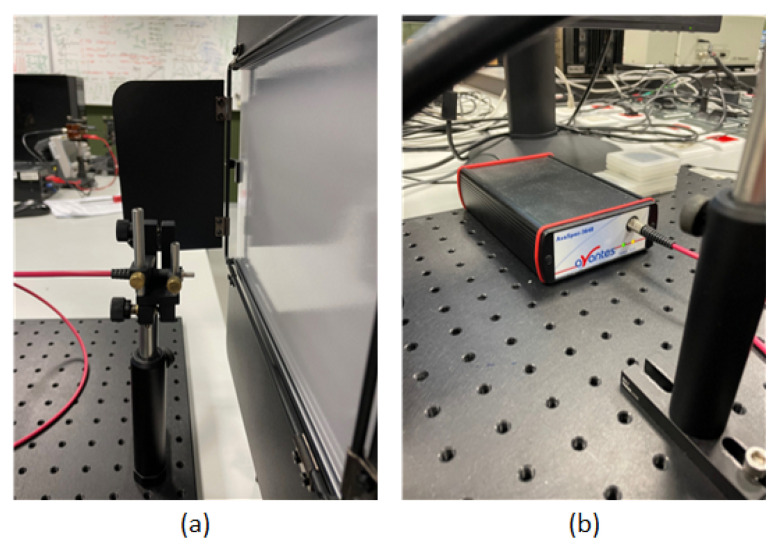
Workstation for measuring the wavelength spectrum of light: (**a**) probe for measurement, (**b**) spectrometer.

**Figure 6:**
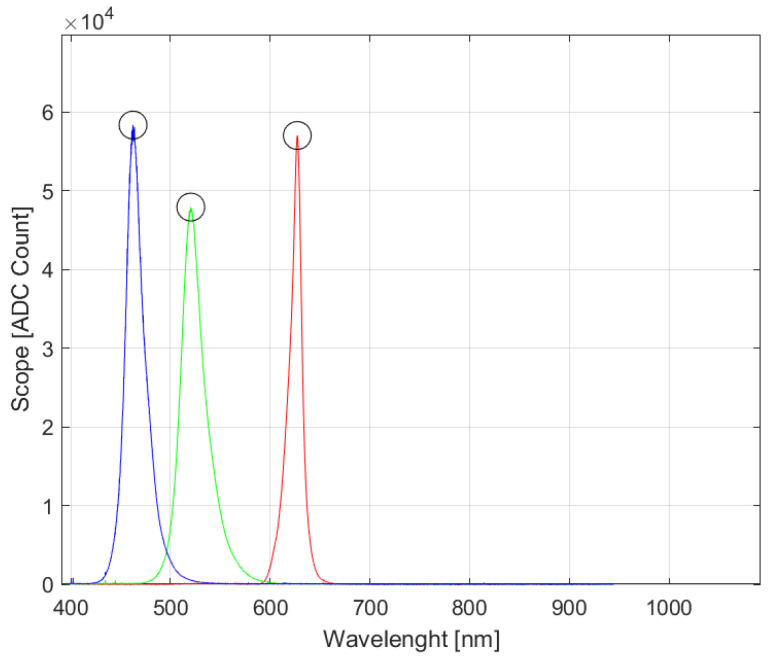
Measurement results of the color spectrum.

Modifying the spectral characteristics of the light source enables the implementation of alternative processes and sensing options, which in turn extends the time required for the creation of the dataset in comparison to the standard method. This approach offers a novel method for enhancing the photogrammetric procedure, which is currently being investigated in various forms [18,19,20]. To ascertain the spectral characteristics of the lighting apparatus utilized (Figure 5a), the Avaspec-3648 spectrometer (Avantes, Apeldoorn, The Netherlands) was employed for precise measurement (Figure 5b). White light encompasses all wavelengths, but by dividing it into its constituent basic wavelengths (red, green, and blue) for subsequent image data preprocessing, more data were obtained. The measuring probe (Figure 5a) was positioned in front of the light source (Figure 6), and the room was rendered completely dark to ensure that the measurement values were not influenced by ambient light.

The experiment yielded the dominant wavelengths of the lights. The red LEDs produced light with a dominant wavelength of 627 nanometers (nm) (R627), the green LEDs with a dominant wavelength of 520 nm (G520), and the blue LEDs with a dominant wavelength of 462 nm (B462). Additionally, the impact of alterations in the spectral characteristics of light on the generation of the 3D model was examined. Modifying the color of the light opens avenues for further inquiry into the creation of 3D models of specific objects with distinctive surface granularity and coloration.

#### 2.2.3. Camera and Equipment Configuration

In the process of creating datasets, a number of parameters can be adjusted during the capture phase. These parameters include factors such as exposure time, aperture settings, ISO sensitivity, white balance, focus distance, and lighting conditions. Each of these parameters plays a crucial role in determining the quality and accuracy of the captured images, which directly impacts the effectiveness of the subsequent 3D model creation. To ensure that the parameters are correctly set, it is essential to implement a small set of test images for rapid verification. This practice allows for the identification and correction of any potential issues early in the process, ensuring optimal settings before proceeding with the full dataset capture. Test images help to fine-tune parameters such as (standard parameters):

1. Exposure time: Adjusting the exposure time ensures that the images are neither overexposed nor underexposed, capturing the correct amount of light. This is particularly important for capturing the intricate details of the butterfly’s wings without losing information in highlights or shadows.
2. Aperture settings: The aperture size affects the depth of field in the images. For macro-photography, a smaller aperture (higher f-number) provides a greater depth of field, ensuring that the entire surface of the butterfly’s wings is in sharp focus. However, it also requires sufficient lighting to compensate for the reduced light entering the camera.
3. ISO sensitivity: Setting the appropriate ISO sensitivity is crucial to minimize noise in the images while maintaining adequate brightness. Lower ISO values are preferred for higher image quality, but they may require longer exposure times or brighter lighting.
4. White balance: Correct white balance settings ensure that the colors in the images are accurately represented. This is essential for maintaining color fidelity, especially when dealing with the iridescent wings of the butterfly, which exhibit a range of colors depending on the viewing angle.
5. Focus distance: Accurate focus is vital for capturing the fine details of the butterfly’s wing scales. Macro lenses with manual focus control are often used to achieve the necessary precision.
6. Lighting conditions: As discussed previously, diffuse lighting from LED sources with diffuse glass helps to reduce shadows and evenly illuminate the butterfly’s surface. The intensity and angle of the lighting can be adjusted to highlight specific features and ensure uniform illumination.

In the case of the selected object, the butterfly *Papilio blumei*, the following parameters were used: resolution of camera, aspect ratio, aperture, exposure time, ISO, and focal length. These parameters were carefully chosen based on preliminary tests and the specific requirements of capturing the butterfly’s detailed and iridescent wing patterns (set parameters in our experiments):

1. Resolution of camera: The camera’s resolution of 6240 × 4160 pixels (26.2 Mpix) ensured that the images had sufficient detail to capture the fine structure of the butterfly’s wing patterns.
2. Aspect ratio: an aspect ratio of 4:3 was chosen to maximize the use of the sensor area, providing a balanced frame that suited the dimensions of the butterfly’s wings.
3. Aperture: A small aperture of F/22 was used to achieve a large depth of field, ensuring that the entire wing surface was in sharp focus. This is particularly important for macro-photography where the depth of field is naturally shallow.
4. Exposure time: An exposure time of 0.8 s was selected to gather enough light for a clear image without motion blur. The camera is typically mounted on a stable platform to avoid any movement during the long exposure.
5. ISO: Setting the ISO to 100 minimized noise, resulting in cleaner and sharper images. Low ISO settings are preferred in controlled lighting environments to maintain high image quality.
6. Focal length: A focal length of 50 mm provided an ideal balance between magnification and working distance. This focal length was suitable for capturing the butterfly at a close range while maintaining a comfortable distance for lighting and manipulation.

**Figure 7:**
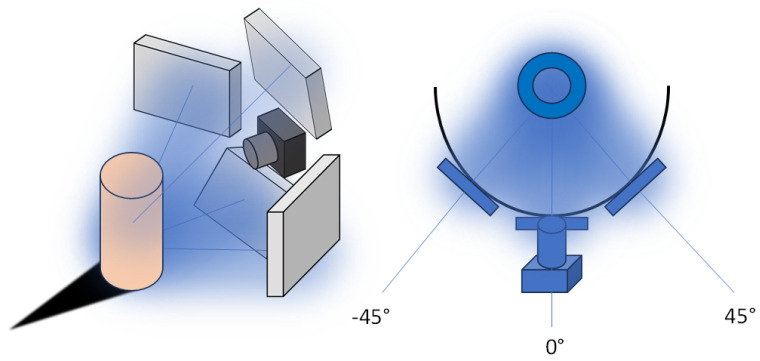
Example of the geometry positioning.

Implementing a small set of test images with these parameters ensured that any adjustments needed could be made swiftly, optimizing the capture settings before the full dataset was acquired. This process not only saved time but also enhanced the quality and consistency of the final dataset, leading to more accurate and reliable 3D models of the butterfly. The datasets were created using the precision object positioning platform, RGB lighting, a Canon 6D Mark II camera (Canon, Tokyo, Japan), appropriate geometric positioning (Figure 7) of the lights, and a powerful computer. This combination of equipment and settings was carefully selected to ensure the highest quality data capture and processing for the 3D modeling of the butterfly *Papilio blumei*.

The Bayer filter in digital cameras influences the separation of RGB (red green blue) channels by limiting the spectral response to only three broad color ranges. This process, while effective for general photography, introduces limitations when applied to spectral imaging for 3D modeling. The interpolation required for color reconstruction can reduce accuracy, leading to artifacts, imprecise reflectance, and a loss of fine surface detail. While spectral imaging can improve 3D models, the inherent limitations of the Bayer filter must be accounted for, particularly in applications where accuracy and fine surface texture are crucial. Potential limitations for 3D models include the following:

1. Color inaccuracy: Since the Bayer filter approximates the full-color image by interpolating data, the final colors in the 3D model can be less accurate, particularly when working under different lighting conditions or with objects that have subtle color variations. These inaccuracies can distort the appearance of surface textures or reflectance properties, affecting the visual realism of the 3D model.
2. Loss of fine detail: In areas with high spatial detail or sharp color boundaries, the interpolation process (demosaicking) can introduce artifacts, such as blurring or false color edges. This is especially problematic for applications where precise surface details are critical, such as cultural heritage preservation or industrial inspection. The Bayer filter’s design prioritizes luminance (brightness) accuracy over color precision, which may not capture intricate patterns or fine textures as effectively.
3. Reduced spectral range: The Bayer filter’s limited spectral response to only RGB light means that it misses out on other parts of the light spectrum (such as ultraviolet or infrared). This limitation is a concern for applications that require more detailed spectral data to differentiate between materials or surface properties. A hyperspectral or multispectral camera would be more appropriate for capturing full-spectrum data, but these technologies are not typically used in standard digital cameras equipped with Bayer filters.
4. Dependence on lighting conditions: The accuracy of the 3D model is highly dependent on the type of lighting used. If the light source is not well-balanced or if different colored lights (e.g., red, green, or blue) are used, the Bayer filter will react differently to each. This leads to an uneven capture of surface details, where certain colors may dominate or wash out other details, particularly if one light spectrum is favored over another. Under monochromatic light, the sensor’s performance can drop significantly since only one color channel is actively contributing to the image, resulting in less accurate models.
5. Surface reflectance and texture limitations: Accurate surface modeling in 3D is dependent on correctly capturing how light interacts with the surface of an object. The Bayer filter’s limited spectral response may not fully capture the reflectivity or texture of materials, especially under complex lighting conditions. This impacts the depth and realism of the 3D models, particularly for objects with intricate surface features or variable reflectance properties.

The precision object positioning platform was crucial for ensuring that the butterfly remained stable and accurately positioned during the imaging process. This platform allowed for fine adjustments in the orientation and placement of the butterfly, facilitating the capture of images from multiple angles while maintaining consistent alignment. Such precision is essential for creating datasets that are free from distortions and misalignments, which could otherwise compromise the accuracy of the 3D model.

RGB lighting was used to provide consistent and adjustable illumination that enhanced the visibility of the butterfly’s iridescent colors. The RGB lights could be tuned to different wavelengths to highlight specific features and reduce color casting, ensuring that the true colors of the butterfly were captured accurately. The geometric positioning of the lights was carefully arranged to eliminate shadows and provide even lighting across the entire surface of the butterfly, as depicted in Figure 7. This setup helped to reveal the intricate details of the wing scales and patterns without introducing glare or reflections.

The Canon 6D Mark II camera, equipped with a Canon EF 50 mm F1.8 STM lens was chosen for its high resolution and excellent optical performance. The camera’s settings, including a resolution of 6240 × 4160 pixels, an aperture of F/22, an exposure time of 0.8 s, and an ISO of 100, were optimized for capturing high-detail macro images. The 50 mm lens provided a suitable focal length for close-up photography, allowing for a detailed and clear representation of the butterfly’s wings.

A powerful computer was used to handle the processing and storage of the large datasets generated during the imaging process. The specifications of the used computer included the following:

1. Processor: The Intel Core i5-13400F 13th Generation processor provided the necessary computational power to handle complex image processing tasks quickly and efficiently. This ensured that large datasets could be processed in a timely manner, facilitating the creation of high-quality 3D models.
2. Graphic card: The NVIDIA GeForce RTX 3080 (NVIDIA, Santa Clara, CA, USA) with 10 GB of memory was used to accelerate rendering and other GPU-intensive tasks. This powerful graphics card significantly reduced the time required for tasks such as image stitching, 3D reconstruction, and rendering, enabling real-time feedback and faster workflow.
3. RAM: With 64 GB of DDR4 RAM, the computer could manage large datasets and multitask effectively. Sufficient memory is crucial for handling high-resolution images and performing complex calculations without bottlenecks.

The integration of these technologies ensured that data capture and processing workflows were optimized for efficiency and accuracy. The combination of precise positioning, high-quality imaging, and robust computing power enabled detailed and accurate modeling of the *Papilio blumei* butterfly, capturing its unique features and iridescent coloration in a realistic 3D representation. The images were created in the standard JPEG format, which is commonly used for imaging. In the camera settings, the uncompressed JPEG format was selected to capture the full image resolution of 6240 × 4160. In previous experiments, the RAW C2R format was employed, but the handling of such large quantities of data proved impractical for the objective of optimizing input data.

#### 2.2.4. Image Capture Process

**Figure 8:**
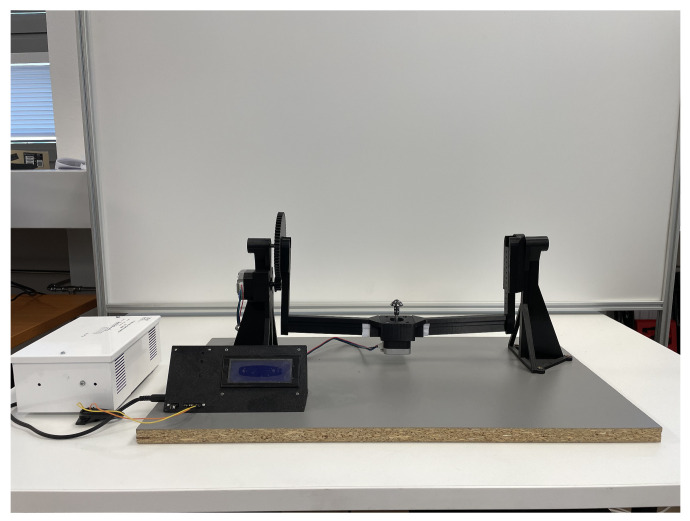
A sensing platform that provides precise positioning of the subject with the option of automatic sensing via remote trigger.

In this work, four datasets were created (each containing 100 images). These datasets were designed to capture comprehensive visual information about the butterfly *Papilio blumei* from multiple angles and lighting conditions, providing a robust basis for 3D modeling. The initial dataset was created using white light, with the object positioned on a sensing platform as illustrated in Figure 8. The use of white light ensured that the full spectrum of visible light was evenly distributed over the object, providing a neutral baseline for capturing the butterfly’s true colors and structural details. This was essential for accurately documenting the iridescent properties of the butterfly’s wings, which can exhibit dramatic color shifts under different lighting conditions. The number of frames per position, the rotation angle, and the number of view planes to ensure comprehensive coverage of the object were specified (Table 3). These parameters are critical to achieving a detailed and accurate 3D reconstruction.

The precision object positioning platform used in this process ensured that the butterfly remained stable and accurately aligned throughout all imaging sessions. This stability was critical for achieving consistent results and avoiding artifacts caused by movement or misalignment. By methodically controlling imaging parameters and systematically varying lighting conditions, datasets were generated that captured the true essence of *Papilio blumei* and provided valuable insights into its unique visual properties.

By adjusting the parameters on the sensing platform, 100 frames were obtained. The images included the front, rear, and profile views of the object. However, the profile images were unsuitable for reconstruction due to the object’s negligible depth, which made it challenging for existing algorithms to identify correspondence points for alignment. The set of images was decomposed into the aforementioned view planes, each containing regions of varying colors. The images were manually sorted according to photogrammetric criteria, with those deemed unsuitable for the alignment process identified and presented in Table 4.

The remaining datasets were generated using distinct spectra of visible light, specifically the standard RGB wavelengths. The emitted light was quantified using a spectrometer, with the values defined as follows: red light with a dominant wavelength of 627 nm, green light at 520 nm, and blue light at 462 nm. The distribution of these datasets was identical to that observed with white light. As the object was imaged on a controlled sensing platform, the position of all images in all datasets remained constant.

#### 2.2.5. Creation of Point Cloud for Spectral Imaging

The objective of creating the dataset for spectral imaging of objects in basic spectra of white light was to provide a comprehensive and diverse collection of spectral images for the purpose of analyzing the impact on 3D models with surface reflectivity. This dataset provided a foundation for understanding how spectral imaging could enhance the accuracy and detail of 3D models, particularly with regard to surface reflectivity. Subsequently, the generated datasets were employed in the construction of 3D models, with the objective of investigating the impact of light parameters on 3D model formation. The three-dimensional models were created using the Agisoft Metashape software program. The generation of a point cloud revealed initial values that exhibited discrepancies in the number of points. These discrepancies were attributable to the light that was incident on the object’s surface, which resulted in disparate representations at disparate wavelengths for the same imaged point positions.

**Figure 9:**
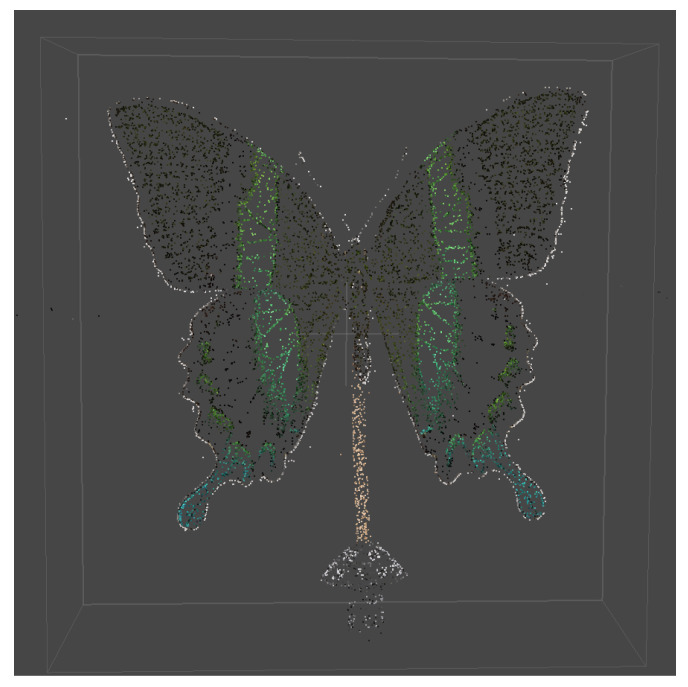
Example of sparse point cloud.

**Figure 10:**
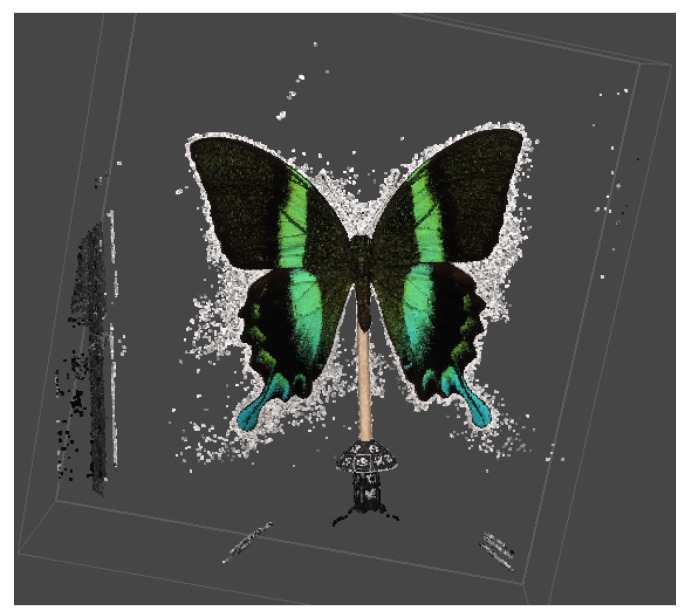
Example of dense point cloud.

The Sparse Cloud Process (Figure 9) involves the alignment of photographs to create connecting points based on the identification of objects in the images. The alignment process yielded the number of points defined for each dataset, as shown in Table 5. However, a more effective visualization was achieved by creating a dense point cloud (Figure 10).

In this process, the internal parameters of the camera were employed to perform a geometric transformation, also referred to as rectification, and to compute epipolar lines, as previously described in the literature, namely, in [21,22]. This transformation was contingent upon the orientation parameters of the camera, which were defined in relation to tie points. Subsequently, an algorithm was employed to assign pixels to pixels in pairs of given frames. This process also encompassed distortion and camera position parameters, as outlined in [23]. Once the precise camera position was determined, a dense point cloud (Figure 10) could be generated, which contained a greater number of points (Table 6) than a sparse point cloud.

Additionally, the generated dense point cloud exhibited a considerable number of superfluous points, which resulted in the formation of artifacts. It was therefore necessary to eliminate these artifacts through filtering based on confidence points. The removal of inaccurate or unreliable points facilitated the enhanced analysis and visualization of the 3D model. Reliability points were calculated from depth maps and transformed into a dense point cloud. A transformation was then applied to the depth map, whereby it was converted into a three-dimensional point cloud utilizing a set of projection equations. Each point in the point cloud was defined by its position in the depth map and its assigned depth, as indicated by the *x*, *y*, and *z* coordinates. The analysis of these points was based on an evaluation of the data quality, specifically the accuracy of the depth points in different image parts. For instance, regions exhibiting low texture or high glare may be identified as points of lesser reliability. The reliability of the data was evaluated through the application of error metrics, which defined the standard deviation or variability of the depths. This process, referred to as data consistency in space, served to eliminate unreliable points, thereby enhancing the overall quality of the 3D model [24,25].

**Figure 11:**
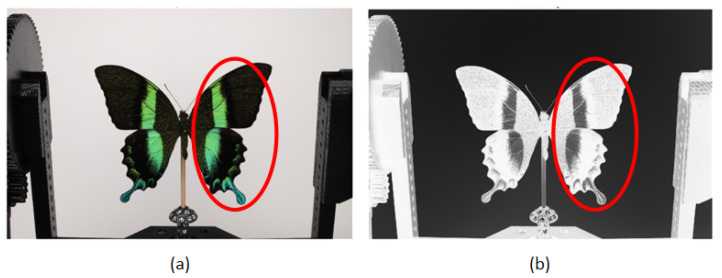
Visualization of depth map for points by confidence: (**a**) original image (**b**) depth map.

Figure 11b illustrates the calculation of the depth map. By representing the intensity (brightness) of an original Figure 11a, it was possible to gain a fundamental understanding of the depth from a single image. The position of the object was indicated by the intensity level. An elevated intensity value signified that the area in question was situated in closer proximity, whereas a diminished value indicated that it was located at a greater distance. Nevertheless, this assumption did not ascertain the precise depth of the object, as evidenced by the delineated (red) region in Figure 11. As illustrated in Figure 10, the generated dense point cloud exhibited the presence of artifacts that necessitated filtering. Such artifacts may include noise, outliers, and inaccuracies in the point cloud data, which may result from a number of factors, including sensor noise, imperfect alignment, or variations in surface reflectivity. The presence of these artifacts can have a markedly deleterious effect on the quality of the 3D model, resulting in inaccuracies and a loss of detail. The application of confidence point filtering enabled the elimination of these artifacts through the assignment of a confidence value to each point in the cloud, based on its reliability and consistency with surrounding points. This method facilitated the isolation and removal of points that were likely to be erroneous, thereby enhancing the quality of the dataset. The selected filtering level for all datasets was a dimensionless value ranging from 0 to 255, which was set to 9. This particular filtering level was selected based on preliminary tests which sought to achieve an optimal balance between the removal of artifacts and the preservation of important surface details.

**Figure 12:**
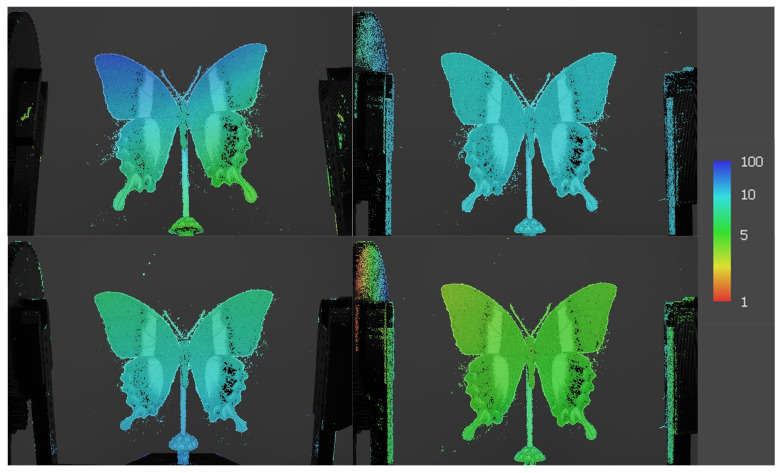
Displaying depth maps from a dataset of images captured in white light used in the experiment. Blue color indicates high reliability, while red indicates low reliability (observable on the scale in percent).

After the filtering method was applied, areas with undesirable characteristics, namely, the presence of “holes” in the point cloud, became apparent. The aforementioned holes represented regions where data points were removed on the grounds of low confidence values, thereby disclosing lacunae in the surface representation. Gaps may result from a number of factors, including occlusions, regions of low reflectivity, or constraints in imaging angles. The generation of holes in the point cloud is a complex phenomenon that is influenced by a number of factors, including the filtering process that removes low-confidence data points. However, this phenomenon is not merely a consequence of filtering; it is also closely related to fundamental challenges in depth perception and point cloud generation, with light playing a significant role. One of the principal causes of the formation of these holes is occlusion, whereby portions of the object or scene are obscured from the sensor’s viewpoint, thereby preventing the acquisition of depth information in those regions. To illustrate, in a complex object with overlapping surfaces or intricate geometries, specific areas may be obstructed from the sensor’s line of sight, resulting in gaps in the data. In addition to occlusion, the variation in surface reflectivity is another contributing factor. The presence of either excessively reflective or excessively absorptive surfaces can result in inaccurate or incomplete depth readings, as illustrated in Figure 12. The presence of highly reflective surfaces may result in the scattering of the sensor’s signal, potentially leading to the generation of erroneous data points that are subsequently filtered out. Conversely, the use of low-reflectivity surfaces may not return an adequate signal, which could result in the omission of points within the point cloud.

In this context, the influence of lighting conditions is of paramount importance. The perception of depth and surface details by the sensor is contingent upon the quality of light. Inadequate or uneven illumination can result in the formation of shadowed regions, which may impede the sensor’s ability to accurately discern depth information. For example, when areas of the object are in shadow or are illuminated inconsistently, the sensor may interpret these regions as having lower confidence, which may result in their removal and the creation of holes in the point cloud. Furthermore, bright or direct lighting can create glare or reflections that distort the depth data, thereby contributing to inaccuracies and gaps. Imaging angle constraints also have a significant impact on the formation of these gaps. When the sensor is positioned at an angle that is suboptimal with respect to the surface being scanned, it may encounter difficulties in accurately capturing depth information. This limitation is particularly evident in areas with sharp angles or deep recesses, where the sensor’s field of view is restricted, resulting in a loss of detail in those regions.

The decision to filter out low-confidence points was a crucial step in improving the overall quality of the 3D model. Nevertheless, this inevitably resulted in the formation of gaps in the dataset. The presence of these gaps could potentially compromise the fidelity of the 3D reconstruction, as the absence of data points results in incomplete surface representations. To address these deficiencies, additional post-processing techniques, such as interpolation or image inpainting, were employed with the objective of filling in the missing data while preserving the integrity of the model. The generation of voids in the point cloud is a complex phenomenon that can be attributed to a number of factors, including occlusions, variations in surface reflectivity, limitations in imaging angles, and lighting conditions. While filtering low-confidence points enhances the overall quality of the dataset, it also reveals these gaps, necessitating further corrective measures to achieve a complete and accurate 3D model. Proper lighting is of particular importance, as it can either mitigate or exacerbate these issues, depending on the manner in which it interacts with the object and the sensor.

**Figure 13:**
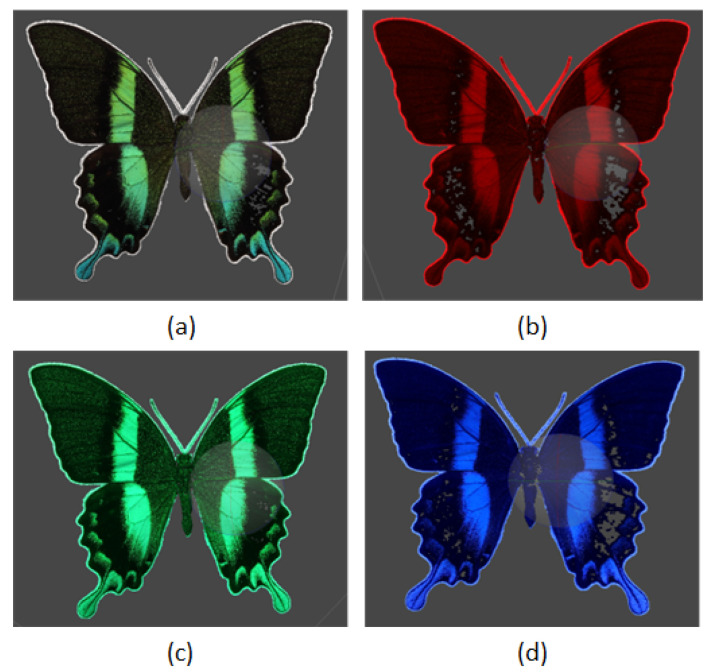
Point clouds and the disparity in errors for individual spectrum: (**a**) white, (**b**) red, (**c**) green, (**d**) blue.

In the remaining datasets RGB, the dimensions of the apertures exhibited variability (Figure 13). This variation could be attributed to the differing reflectance properties of the butterfly’s wings at various wavelengths. To illustrate, specific scales on the butterfly’s wings may reflect red light with greater efficacy than blue or green light, resulting in a greater number of data points and a reduction in the number of holes in the green dataset relative to the blue or red datasets. The inherent structural coloration and iridescence of *Papilio blumei*’s wings contribute to these discrepancies, as the interaction of light with the microscopic scales can vary significantly with wavelength.

#### 2.2.6. Comparison of 3D Models

The 3D models were exported in various formats for evaluation purposes. Therefore, it was necessary to select a standard format for better comparison of the results. The most commonly used formats are .PLY, .OBJ, and .STL. The .STL format was not used as the primary format for comparison methodology. The 3D models considered as reference could be exported in .STL format. This format was later converted into a point cloud to allow further processing. The 3D models were directly exported to the .PLY format, which preserves the resolution of the 3D model, that is, the number of points in the model without any optimization. The methodology for comparing 3D models consisted of several steps, which were maintained for all 3D models:

1. Alignment of the 3D model with the reference model.
2. Automatic function for minimizing alignment error using ICP (Iterative Closest Point).
3. Selection of the area of interest with the greatest defect.
4. Calculation of the absolute difference between 3D models.
5. Processing of the obtained results.

**Figure 14:**
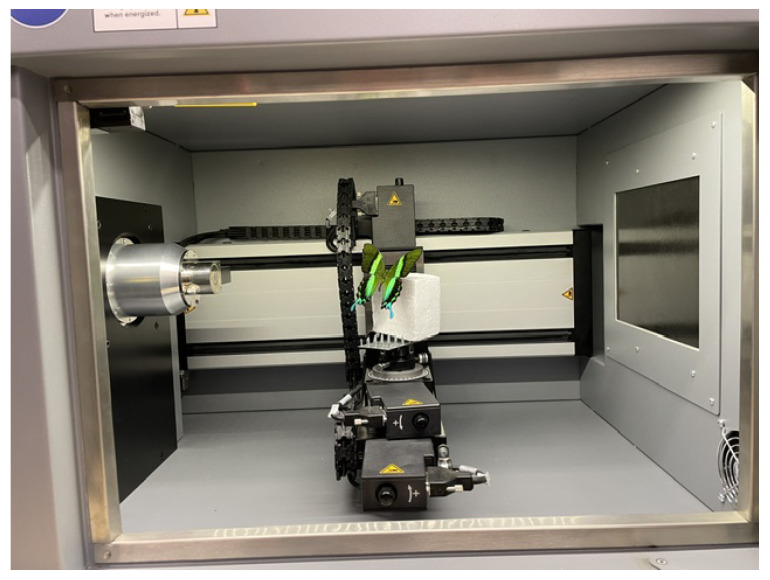
Creating a reference scan (DeskTom CT scanner) [26].

The reference scan was generated using a device designated as DeskTom [26]. The DeskTom device (Figure 14) operates on the principle of digital radiography, whereby the image is formed by the shadow created by the varying X-ray absorption of the object. The absorbed radiation is dependent upon the photon number of the substance composing the object. The object is subjected to irradiation, and the resulting image is subsequently processed. A specialized detector is employed to visualize the shadow image, whereby X-ray photons are converted into an electrical signal. The scanner employs a direct radiography process, enabling the object to be digitally imaged in sections. The dimensions of the detector, which is defined as the active scanning area, impose limitations on the size of objects that can be scanned by the scanner. The scanner’s parameters are presented in Table 7.

**Figure 15:**
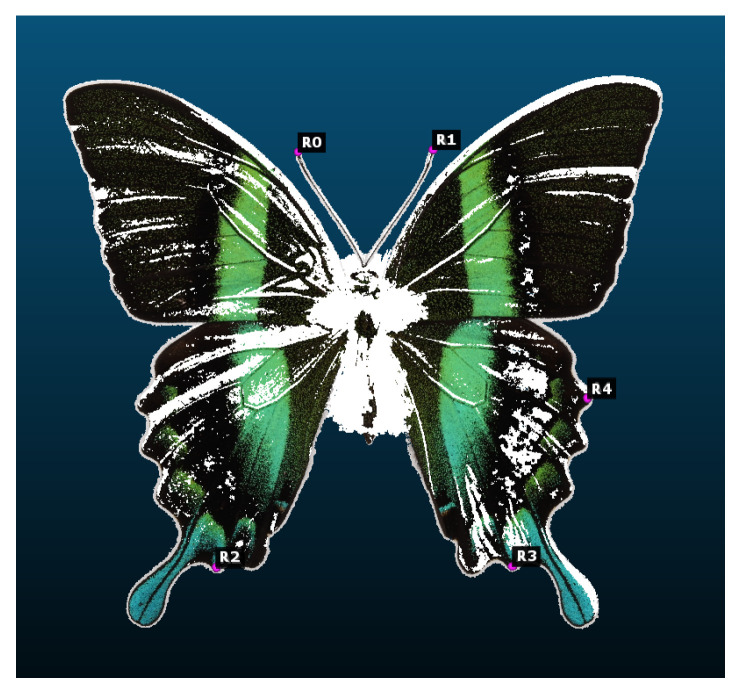
Alignment of the created 3D model with the reference.

The process of aligning 3D models using a manual function was necessary due to the differences in the size of the 3D models and their position in space (rotation, placement, and viewing position). For the alignment of the 3D model, it was necessary to manually place at least five points. The rationale behind the necessity of five points is to guarantee spatial orientation and the selection of the most pertinent areas visible on both the reference 3D model and the compared 3D model. By performing this task, it was possible to align the 3D models, taking into account any constant error that, if further manipulated, would result in the degradation of the output data. The alignment of the two 3D models was performed using the Horn algorithm [27] for registering corresponding points and aligning based on five points in Figure 15.

**Figure 16:**
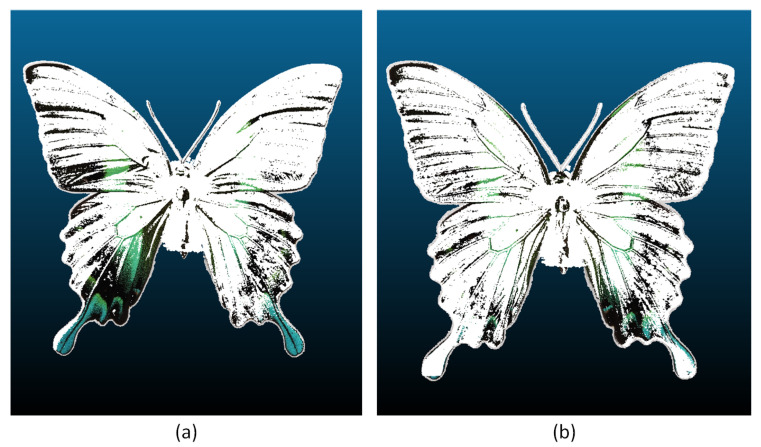
Alignment of the reference 3D model with comparison: (**a**) manual function min. 4 points, (**b**) automatic function using the ICP algorithm.

Nevertheless, despite the meticulous precision involved in manual alignment, the potential for human error to be introduced during this process remains. This is illustrated in Figure 16a. Such discrepancies may result from the inaccurate positioning of the points, which could lead to minor misalignments between the reference model and the model being compared. These manual inaccuracies could result in suboptimal alignment, which would affect subsequent analyses and the overall integrity of the comparison.

In order to mitigate these errors, the process was complemented with the addition of automated alignment techniques, specifically the Iterative Closest Point (ICP) algorithm, which was used in conjunction with Cloud Compare (see Figure 16b). Following the preliminary manual alignment, the ICP algorithm refined the alignment by minimizing the discrepancies between corresponding points on the two models in an iterative manner. This automated process addressed any inconsistencies introduced during the manual alignment, thereby ensuring a more accurate and reliable comparison between the 3D models. Consequently, the integrated approach of manual alignment followed by automated correction provided a robust methodology for aligning 3D models, reducing the probability of errors and enhancing the precision of the final output.

The ICP algorithm uses a process in which the reference 3D model remains unchanged while the rotation, scaling, and translation parameters are modified for the aligned model. ICP is the most commonly used algorithm for point cloud registration and subsequent comparison. The ICP algorithmic process consists of the following steps:

1. Estimate the position of one point cloud relative to the other point cloud.
2. For each point in the first point cloud, find the nearest point in the second point cloud. In this step, the algorithm tries to find the closest points to the corresponding points.
3. Calculate the translation and rotation of the point cloud from the first two steps to minimize the distance and increase the overlap. This step represents the translation and rotation of the point cloud.
4. Repeat the previous steps to minimize and correct the alignment error of the point clouds and improve the overlap.
5. Terminate the alignment process when the number of iterations or the deviation value is reached.

**Figure 17:**
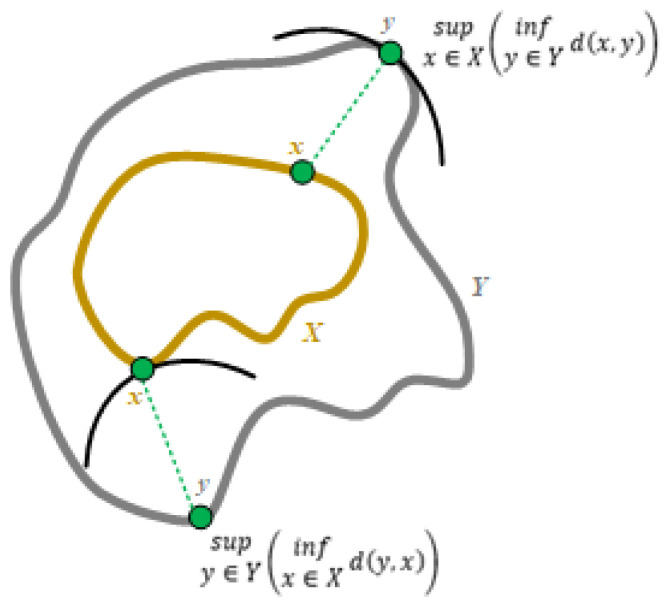
Computation of Hausdorff distance between two lines.

The Hausdorff algorithm [28,29] was employed for the calculation of the absolute distance. This algorithm is employed to compute the distance between two subsets. The objective is to transform the set of non-empty compact subsets of a metric space into the metric space itself. In order to calculate the Hausdorff distance (HD), it is necessary to determine the distance of each point in one set from the nearest point in the other set, and vice versa (Figure 17). This metric is robust for the comparison of sets of points and is applicable in various fields focused on shape or pattern analysis and comparison. To compute the Hausdorff distance (HD), a function was employed that identified the nearest point in the compared three-dimensional (3D) model relative to the reference 3D model. The point clouds were approximately aligned, as a function for aligning 3D models was employed in the preceding step. The distance could be interpreted using the following formula for two sets of points, *X* and *Y*:

(1) H(X,Y)=max{δ(X,Y),δ(Y,X)}

where δ (*X*, *Y*) and δ (*Y*, *X*) are the one-way Hausdorff distances between the sets. These sets can be interpreted as follows:

(2) $$\delta \left(X,Y\right)=\underset{x\in X}{\sup}\left(\underset{y\in Y}{\inf}d\left(x,y\right)\right)$$

(3) $$\delta \left(Y,X\right)=\underset{y\in Y}{\sup}\left(\underset{x\in X}{\inf}d\left(y,x\right)\right)$$

where sup() is the superior bound, and inf() is the inferior bound. To calculate the distance between set *X* and set *Y*, the first relationship was used, measuring the greatest distance. A point in set *X* was selected, and then the nearest point in set *Y* was identified, and the distance between the two points was measured. In the second interpretation, the function reversed the order of the sets (*Y*, *X*) and identified the nearest point in set *X*, thereby indicating the extent to which the points in set *Y* differed from those in set *Y* [29,30].

## 3. Experimental Results

In this section, the achieved experimental results are described. The experimental results demonstrated a significant improvement in capturing fine textures and subtle surface variations, leading to more detailed and nuanced 3D representations. Specifically, in the case of the *Papilio blumei* butterfly, spectral imaging accurately captured the intricate patterns and iridescent colors of its wings, which conventional RGB imaging could not adequately address due to the dynamic color shifts. In summary, the incorporation of spectral imaging data significantly enhanced the precision and complexity of the 3D models, particularly for objects with intricate patterns and diverse material characteristics, such as the *Papilio blumei* butterfly.

### Results

To explore this area and assess error rates within individual datasets across various spectra of visible light, Cloud Compare was utilized as the primary analytical tool. A dense point cloud was used for comparison due to the minimal depth in the visualization of the imaged object, specifically, a butterfly. Consequently, the resulting model was rendered as a two-and-a-half-dimensional (2.5D) representation rather than a full three-dimensional (3D) model. To ensure objective comparisons between the generated 3D models, reference scans were incorporated into the analysis. These reference scans were created using high-precision imaging techniques, providing a baseline to measure deviations and discrepancies in the 3D models generated from spectral imaging data. This comparative analysis not only helped evaluate the impact of spectral lighting on model accuracy but also highlighted areas for improvement in the integration of spectral data within the 3D modeling workflow. By using reference scans, the study ensured a rigorous evaluation of the quality and fidelity of the spectral-based 3D reconstructions.

**Figure 18:**
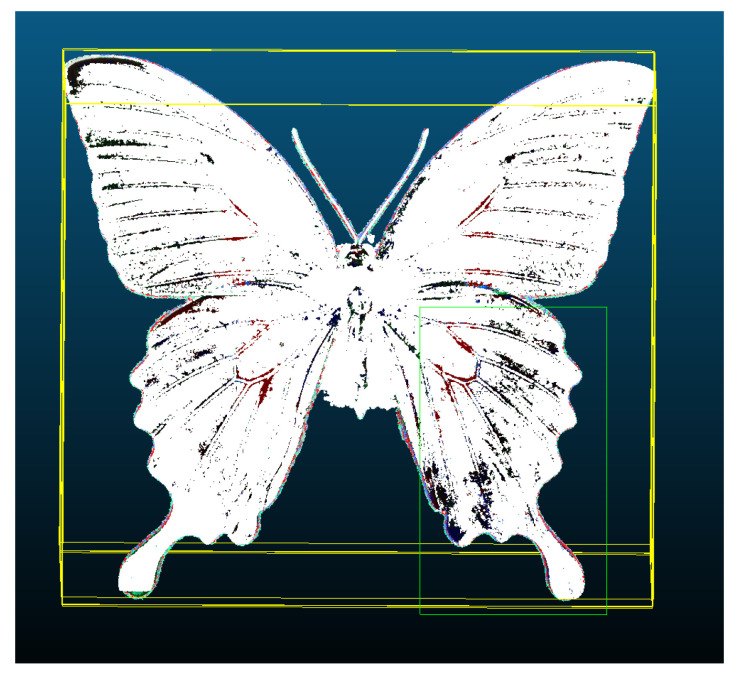
Selecting an area of interest from all point clouds.

**Figure 19:**
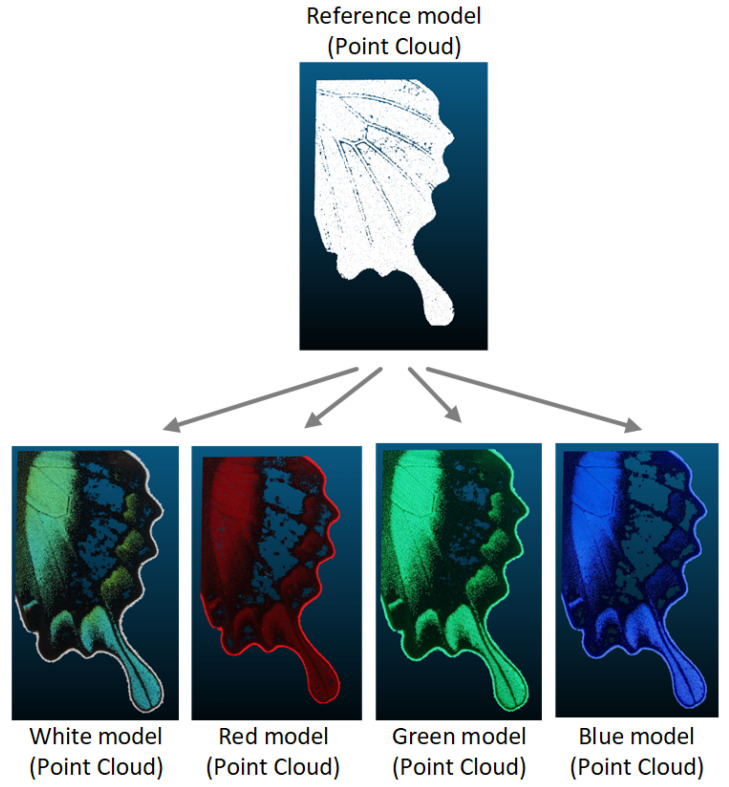
Comparison of generated point clouds with the reference point cloud.

The point clouds were aligned through the placement of a minimum of four points on each cloud. To guarantee uniform alignment, it was necessary for the points to be situated at approximately the same location on each object. As illustrated in Figure 18, the same points were utilized on each object for the sake of simplicity. This process required the placement of five alignment points 16 times. The WRGB (white red green blue) models were aligned with the reference model, which was created with the CT scanner described in the preceding section. This alignment process ensured that the size of the clouds was consistent with that of the reference cloud, thus guaranteeing a 1:1 scale for the models. The region of interest (Figure 18) was selected in a proportional manner from all point clouds in order to ensure consistency and to prevent errors in subsequent processing. The comparison regions were extracted from the remainder of the point cloud and subjected to further processing in order to evaluate the results. The region of interest contained a defect that was consistently present in each of the clouds. Upon closer examination, the black area represented the ideal black color, which interacted with light at a specific wavelength. This phenomenon may also be attributed to errors in the generation of depth maps from individual images, which can introduce inaccuracies into the 3D model creation process. Consequently, in order to ascertain the cause of this defect and to develop a solution, it was necessary to define the absolute distance between the generated point cloud and the reference point cloud and to determine how to eliminate this defect. In order to facilitate a meaningful comparison between regions (Figure 19), it was essential to ensure that the reference point cloud was of a greater size than the point cloud being evaluated. By comparing these values with the visualization presented in Table 8, it was possible to identify the specific locations where discrepancies in point values existed for each dataset.

Subsequently, the segmented regions were subjected to a comparative analysis with a reference region. To complete this process, it was necessary to swap the roles of the individual clouds in order to calculate the absolute distance, which improved the visualization of the data. In the calculation of the absolute distance, a metric was employed whereby the point being compared was the closest point. In order to elucidate the rationale behind the necessity of interchanging the point clouds, it was essential to define the chosen metric. When a point from the reference point cloud was compared with a point cloud that was being evaluated, an issue arose if the closest point to the reference was missing, thereby precluding the possibility of defining the distance or error. By interchanging the point clouds (i.e., comparing the created point cloud with the reference point cloud), it was possible to define the error with greater accuracy. Once the compared reference point cloud (CT scanner) had been matched against the generated point clouds (WRGB), the error could be defined for each point. This methodology guaranteed that no point was without a defined absolute distance, thereby facilitating a more accurate interpretation of the results within the individual comparisons. By reversing the roles of the point clouds (reference and comparison), preliminary distance calculations were obtained, which was then automatically implemented when distances were set.

**Figure 20:**
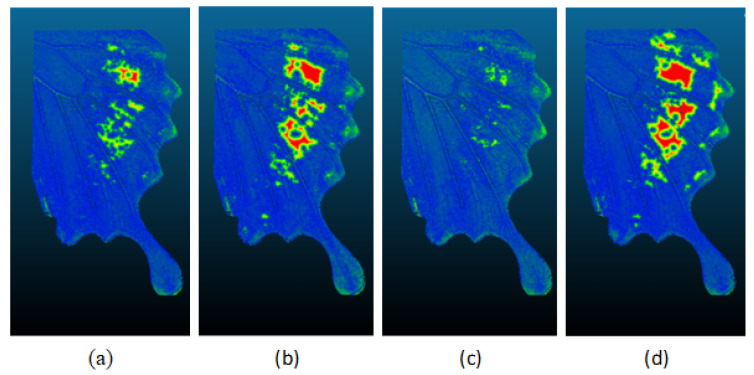
Comparisons of datasets: (**a**) W (white) dataset; (**b**) R (red) dataset; (**c**) G (green) dataset; (**d**) B (blue) dataset.

The Cloud Compare program determined the optimal maximum distance between the point clouds, as shown in Table 9. It then adjusted the predefined distance values, expressed in millimeters, to a uniform value representing a maximum distance of one millimeter. The visualizations for each comparison are shown in Figure 20. In each comparison, it is possible to see which point cloud had the highest degree of error. The point cloud shown in blue had the highest error rate for missing points within the region of interest (Figure 20d). This deficiency could be attributed to the fact that under blue light, black regions could not be effectively registered by the algorithms, in contrast to their performance under other wavelengths of light. In the previous section, the absolute distance between the point clouds in advance was calculated. To ensure consistency in the results of subsequent comparisons, a distance with a maximum value of 1 mm was used (Table 9).

**Figure 21:**
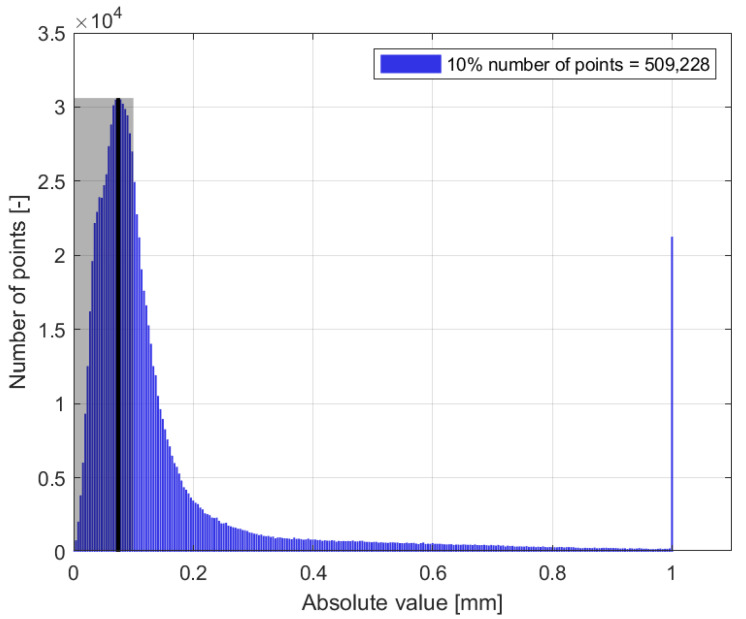
Histogram of number of points versus distance [mm], blue dataset.

**Figure 22:**
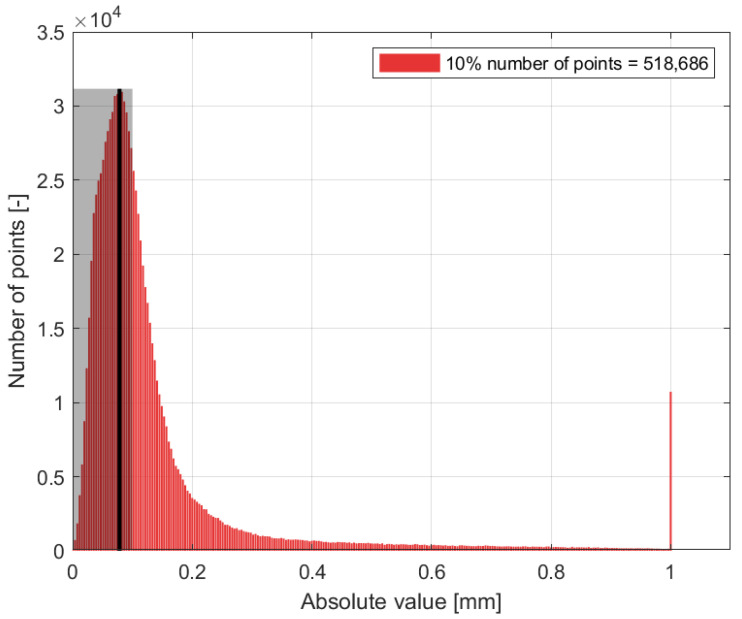
Histogram of number of points versus distance [mm], red dataset.

**Figure 23:**
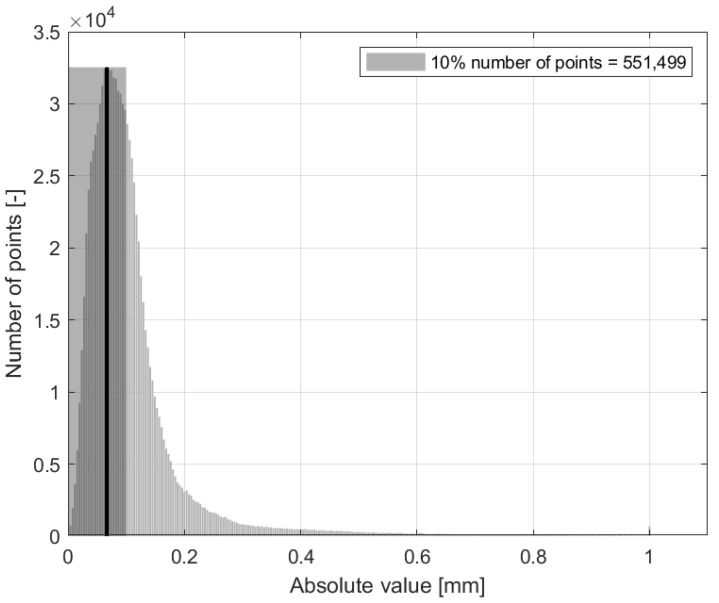
Histogram of number of points versus distance [mm], white dataset.

In the case of the compared region, which contained 978,291 points, the majority of the distances were less than 1 mm, with the majority of these distances ranging from 0 to 0.2 mm. However, in the blue dataset (Figure 20d), red areas indicating distances greater than 1 mm were observed (Figure 21). A comparison of the red dataset, as shown in Figure 22, showed a reduction in the error rate compared to the previous dataset (Figure 21). The majority of the points under comparison had a relatively small distance between them, with this distance decreasing rapidly as the distance between them increased. At a distance of 1 mm, a sharp increase with 10,000 distant points was observed. This discrepancy is clearly seen in Figure 20b. By plotting the absolute distance for the white dataset (Figure 20a) and comparing it to the previous blue (Figure 20d) and red (Figure 20b) datasets, it is observed that the white dataset had smaller distances (Figure 23). The process of creating such a point cloud was appropriate for the integrity and representation of the 3D model. As shown in Figure 20a, the discrepancy is visible in regions where the generated 3D model had less inaccuracy.

**Figure 24:**
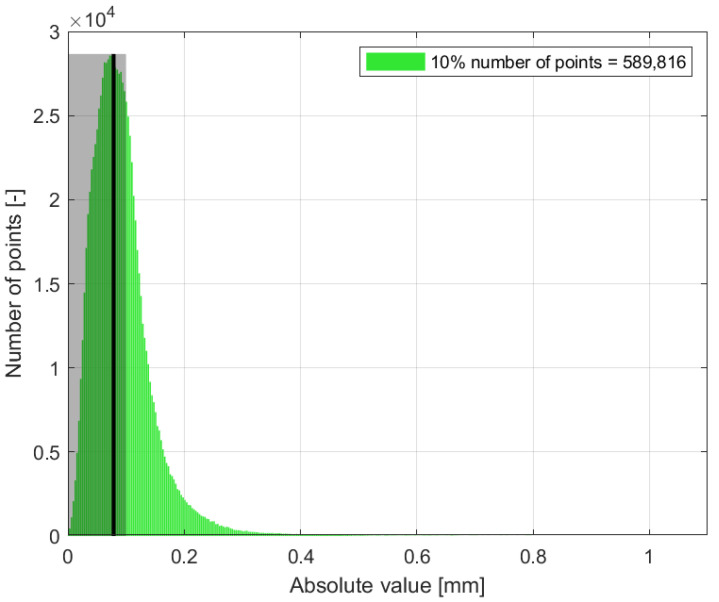
Histogram of number of points versus distance [mm], green dataset.

The final comparison of the absolute distance for the green dataset is represented in Figure 24. It is evident that the distance in the green point cloud decreased and was approaching near-zero values. This discrepancy is evident in the region of interest depicted in Figure 20c). This phenomenon is attributed to the object’s surface, where the green wavelength is in close alignment with the surface structure. For such colored surfaces, green visible light provides a superior option for digitizing this specific surface. The digitization of such surfaces and the utilization of the visible spectrum can effectively eliminate deficiencies caused by surface reflectivity. Future applications of varying wavelengths may yield more optimal results by implementing additional wavelengths beyond the visible light spectrum in digitization applications.

Based on the achieved results (Table 9 and Figure 20), we would like to emphasize that using discrete spectral lighting (white, red, green, and blue) enabled more precise spectral data collection, allowing for enhanced capture of fine surface details and subtle variations that standard RGB imaging may not fully capture. RGB cameras rely on the Bayer filter, which averages the response across broader spectral bands, potentially losing critical information in areas where reflectivity or surface characteristics vary in specific wavelengths. By employing individual colored light sources, our methodology provided additional spectral information, capturing more precise variations in reflectivity across the spectrum. This enhanced the depth and quality of the 3D model, particularly for objects with complex material compositions or iridescent surfaces, such as the butterfly model used in our experiments. Using illumination with discrete white, red, green, and blue (WRGB) light over traditional RGB imaging offered several advantages:

1. Enhanced surface detail: illumination with individual color bands allows for the capture of fine surface textures and subtle variations that may be overlooked in standard RGB imaging, which combines all color channels simultaneously.
2. Targeted spectral data: WRGB illumination isolates specific spectral responses, enabling more focused data collection. This leads to a more accurate representation of surface reflectivity and material properties under each light condition.
3. Improved material differentiation: Different materials reflect and absorb light differently across the color spectrum. By using discrete light sources, it becomes easier to differentiate between materials based on how they interact with specific wavelengths.
4. Accurate color representation: Standard RGB imaging can be limited in accurately capturing dynamic color shifts or iridescent surfaces. Discrete WRGB illumination can more precisely capture such effects, providing a fuller representation of an object’s color properties.
5. Reduction of lighting artifacts: By controlling the lighting conditions and isolating each color band, unwanted reflections and lighting artifacts can be minimized, resulting in cleaner, more accurate 3D models.
6. Greater control in photogrammetry: In 3D modeling, especially with photogrammetry, lighting plays a critical role in image quality. Using separate WRGB light sources allows for more control over illumination angles and intensities, improving the accuracy of the point cloud generation and surface mapping.
7. Broader application flexibility: With the ability to illuminate an object in discrete bands of light, researchers can selectively focus on the wavelengths most relevant to their specific application, whether for cultural heritage, medical imaging, or industrial inspection.

By using WRGB illumination, finer details and color variations can be captured with greater precision, making it a valuable tool for enhancing the quality of 3D models.

## 4. Discussion and Conclusions

The incorporation of spectral imaging into the 3D modeling process, utilizing the fundamental spectra of visible light, has the potential to enhance the accuracy and detail of the resulting 3D models. The experiments demonstrated that 3D models generated under the RGB spectrum exhibited disparate error scales, indicating that multispectral imaging could enhance the quality of 3D models by capturing a more comprehensive surface representation. This study identified several key findings and implications for the field of 3D reconstruction and imaging. One of the most notable outcomes of incorporating spectral imaging was the enhanced representation of surface reflectivity in three-dimensional models. Conventional 3D reconstruction methodologies frequently encounter difficulties in accurately capturing and representing the reflective properties of surfaces, particularly those with intricate textures or varying material properties. The deployment of spectral imaging allows for the capture of detailed information across a range of wavelengths of light, thereby facilitating a more comprehensive understanding of the manner in which surfaces interact with light. This results in the generation of more realistic and visually accurate three-dimensional models. The results of experiments demonstrated that the use of spectral imaging significantly enhanced the visual and geometric accuracy of 3D models. The comprehensive spectral data enabled the capture of nuanced variations in surface texture and reflectivity that were challenging to discern using conventional imaging techniques.

Despite the considerable advancements achieved through spectral imaging, it is important to acknowledge the existence of several challenges and limitations. The primary challenge is the increased complexity and time required for data capture and processing. Spectral imaging necessitates the capture of multiple images under different spectra of light, a process that can be time-consuming and requires precise calibration of the equipment. Furthermore, the incorporation of spectral data into the 3D modeling process necessitates the utilization of advanced computational techniques and algorithms, which may require substantial processing power and expertise. Another limitation is the potential for variation in lighting conditions, which can affect the consistency and accuracy of the captured spectral data. It is of the utmost importance to ensure that the imaging environment is controlled and consistent in order to minimize the aforementioned variations and to achieve reliable results. The integration of spectral imaging under basic spectra of visible light into 3D modeling significantly enhances the accuracy and detail of 3D models, particularly in terms of surface reflectivity. While this approach presents certain challenges and limitations, the benefits it offers in terms of enhanced visual and geometric accuracy are considerable. Future research should concentrate on optimizing the integration process and investigating the potential of this technology in a variety of fields. The integration of macro-photogrammetry with spectral imaging entails the capture of surface reflectivity properties under a range of spectra of visible light. Spectral imaging captures the manner in which different wavelengths of light interact with the object’s surface, thereby providing comprehensive data on the reflectivity and material characteristics of the object. The combination of geometric accuracy obtained from photogrammetry with detailed surface information from spectral imaging results in a more holistic representation of the object. This enhanced model not only depicts the object’s shape but also its reflective properties, rendering it particularly valuable for applications requiring high-fidelity surface details, such as cultural heritage preservation, industrial inspection, and scientific research.

The novelty of this paper lies in the integration of spectral imaging with traditional 3D modeling techniques to significantly enhance the accuracy, surface detail, and reflectivity of 3D models. While traditional RGB imaging methods are limited in their ability to capture fine textures and dynamic surface variations, this research leveraged spectral imaging across different wavelengths, offering a comprehensive approach that captured subtle surface features and material properties. This innovative method is particularly effective for objects with complex surface patterns, such as those in cultural heritage preservation and scientific research, making it a unique contribution to the field of 3D modeling. Additionally, the use of spectral data provides a more accurate representation of surface reflectivity, a critical factor in producing reliable 3D models for applications requiring high precision. This combination of spectral imaging and 3D modeling addresses a significant gap in traditional methods, advancing the state of the art in generating highly detailed and realistic 3D representations. In other words, this approach opens up new possibilities for multidisciplinary research and technological applications, with future studies potentially optimizing the process further to make spectral imaging a standard technology across many scientific and industrial fields. The findings of the study underscore the substantial advantages of incorporating spectral imaging data into the generation of three-dimensional models, particularly in the context of enhancing surface intricacy and reflectivity. Spectral imaging captures images at multiple wavelengths, with separate captures for the red, green, and blue (R, G, B) channels, providing a more comprehensive dataset than is possible with traditional capturing.

Future research should focus on optimizing the integration of spectral imaging data into three-dimensional modeling workflows. This could involve developing more efficient algorithms for processing spectral data, as well as incorporating advanced spectral analysis techniques to further improve model accuracy and detail. Additionally, the application of machine learning and artificial intelligence could automate and streamline the spectral imaging process, significantly reducing the time and effort required for data capture and processing. Research could also explore the use of AI to enhance the accuracy of object identification, surface feature recognition, and reflectivity analysis within spectral datasets. Furthermore, future studies should investigate the potential of combining spectral data with other imaging modalities, such as laser scanning or multispectral imaging, to create even more comprehensive and accurate 3D models, particularly for complex or high-precision applications in fields like cultural heritage preservation, industrial inspection, and biomedical research.

## Figures and Tables

**Table 1:** Occurrence of light parameters.

Parameter	Value
Power of light	10%
Color temperature	5600 K

**Table 2:** Specification of the lights used.

Parameter	Value
Model	RED Head RGB-60
Power	60 W
Number of LED diodes	600 pieces
Luminosity	6000 lm
CRI	95+

**Table 3:** Positioning settings of the object on the sensing platform.

Parameter	Number
Number of frames	32
Angle of rotation	25
Number of view planes	3

**Table 4:** Image layouts from the extracted dataset.

Parameter	Number
Total number of frames	100
Front view plane	45
Rear view plane	44
Inappropriate images	11

**Table 5:** Number of point clouds from the created datasets.

Dataset [Color]	Number of Points [-]
White	14,843
Red	18,225
Green	14,559
Blue	17,245

**Table 6:** Dense point cloud for the datasets.

Dataset [Color]	Number of Points [-]
White	1,315,903
Red	1,237,216
Green	1,343,033
Blue	1,239,863

**Table 7:** DeskTom scanner parameters.

Parameter	Value
Detector resolution	1920 × 1536 pixels
Active area	195 × 244 mm
Imaging	1–60 [FPS]
Color depth	16 bit (65,536 levels of gray)
Pixel size	127 µm
Resolution	5 µm
Maximum dimensions of the scanned object	180 mm (diameter) × 230 mm (Heights)

**Table 8:** Number of points from segmented areas for each dataset.

Name of the Point Cloud	Number of Points [-]
Reference scan	978,291
White	224,790
Red	203,917
Green	239,822
Blue	202,224

**Table 9:** Calculated error values in Cloud Compare.

Name of the Point Cloud	Maximum Distance [mm]
White vs. reference	1.236
Red vs. reference	1.916
Green vs. reference	0.784
Blue vs. reference	2.106

## Data Availability

The data presented in this study are available on request from the corresponding author. This is according to the laboratory rules.

## Abbreviations

The following abbreviations are used in this manuscript:

2D	Two dimensional
3D	Three dimensional
AR	Augmented Reality
CT	Computed Tomography
ICP	Iterative Closest Points
LED	Light Emiting Diode
LiDAR	Light Detection and Ranging
MVS	Multi-View Stereo
PLY	Polygon File Format
RGB	Red Green Blue
SfM	Structure from Motion
SIFT	Scale-Invariant Feature Transform
SURF	Speeded-Up Robust Features
ToF	Time of Flight
UAV	Unmanned Aerial Vehicle
WRGB	White Red Green Blue
